# I aim to specialise in acute paediatric mental health; why isn't psychiatry the obvious choice for training?

**DOI:** 10.1192/bjb.2023.81

**Published:** 2023-12

**Authors:** Rajaei K. Sharma

**Affiliations:** RCPsych Foundation Fellow, FY1 Doctor, Royal Berkshire Hospital, UK. Email: rajaei.sharma@nhs.net

Given the theoretically large overlap in care provided by psychiatrists and paediatricians, young people's mental health should be exceedingly well provided for and managed. However, we know of nationally chronic provision issues in child and adolescent mental health services (CAMHS) and a disconnect between mental health and physical healthcare provision across paediatric services. Many still claim that ‘paediatricians are the last real generalists’.^1^ If that generalism doesn't encompass mental health, is anyone a generalist anymore?

Through placements and medical jobs, specific cases stay with you. Across the course of several medical school placements in paediatrics, those individual cases had one common theme – a mental health component. Whether it was the remarkable attention to detail in the case of a 12-year-old admitted with severe gastroenteritis who had a history of self-harm, or the occasion where a covering consultant simply ‘didn't know what to say’ to a 10-year-old on their first admission with an eating disorder, the commonality between these cases was that the mental health factor stood out like a beacon to every member of staff like no other comorbidity or patient-specific factor.

During my first job as an FY1 doctor, I experienced this challenge of care in a much more acute capacity. Working in an acute psychiatric hospital, I was asked to assess a 15-year-old girl with a head injury. She was being held in the ‘place of safety’, a temporary facility for acutely unwell patients pending transfer to an appropriate ward (in this case, out of area). Owing to several episodes of headbanging, the patient had a severe head swelling, bilateral racoon eye bruising and unequal pupils on my assessment. In any other case, whether paediatric or adult medicine, this would be someone deemed to require urgent medical assessment. But owing to the compounding mental health factor, who did the duty of care fall to? A lack of coherence between the medical and psychiatric teams became evident extremely quickly. I was told that the risk of transfer was a psychiatric decision but then in rebuttal was told that the urgency or need for transfer was purely a medical decision. It was not a straightforward evening.

In adult medicine, a stroke team is more than well equipped to manage a patient's concurrent pneumonia. On a paediatric ward, the generalists that are the paediatricians are easily able to manage a patient's diabetic ketoacidosis alongside an asthma exacerbation. But when it comes to mental health, we see that this ability to manage, at least acutely, lags. There is change on the horizon. The Royal College of Paediatrics and Child Health have introduced a SPIN module for mental health which simply offers some structured additional training.^2^ The Royal College of Psychiatrists currently has a run-through pilot for CAMHS, which has been running since 2018.^3^ My personal ambition is to specialise in acute paediatric mental health – where do I go?
